# Genetic and Phenotypic Evidence for the Causal Relationship Between Aging and COVID-19

**DOI:** 10.1093/geroni/igab046.1283

**Published:** 2021-12-17

**Authors:** Ranran Zhai, Timothy Pyrkov, Anastasia Shindyapina, Marco Mariotti, Peter Fedichev, Xia Shen, Vadim Gladyshev, Kejun Ying

**Affiliations:** 1 Sun Yat-Sen University, Guangzhou, Guangdong, China (People's Republic); 2 Gero, Singapore, Not Applicable, Singapore; 3 Brigham and Women's Hospital, Harvard Medical School, Brigham and Women's Hospital, Harvard Medical School, Massachusetts, United States; 4 Brigham and Women's Hospital, Boston, Massachusetts, United States; 5 Harvard University, Boston, Massachusetts, United States

## Abstract

Epidemiological studies revealed that the elderly and those with comorbidities are most susceptible to COVID-19. To understand how genetics affects the risk of COVID-19, we conducted a multi-instrument Mendelian Randomization (MR) analysis and found that the genetic variation that supports a longer life is significantly associated with the lower risk of COVID-19 infection, as well as being hospitalized after infected. The odds ratio is 0.31 (P = 9.7e-6) and 0.46 (P = 3.3e-4), respectively, per additional 10 years of life. We further applied aging clock models and detected an association between biological age acceleration and future incidence and severity of COVID-19 infection for all subjects and individuals free of chronic disease. Biological age acceleration was also significantly associated with the risk of death in COVID-19 patients. A bivariate genomic scan for age-related COVID-19 infection identified a key contribution of the Notch signaling pathway and immune system. Finally, we performed MR using 389 immune cell traits as exposure and observed a significant negative correlation between their effect on lifespan and COVID-19 risk, especially for B cell-related traits. More specifically, we discovered the lower CD19 level on B cells indicates an increased risk of COVID-19 and potentially decreases the lifespan expectancy, which is further validated in clinical data from COVID-19 patients. Our analysis suggests that the factors that accelerate aging and limit lifespan cause an increased COVID-19 risk. Thus, the interventions target these factors (e.g., reduce biological age), after further validation, may have the opportunity to reduce the risk of COVID-19.

